# Side matters: differences in functional outcome and quality of life after thrombectomy in left and right hemispheric stroke

**DOI:** 10.1186/s42466-022-00223-7

**Published:** 2022-11-21

**Authors:** Milani Deb-Chatterji, Fabian Flottmann, Lukas Meyer, Caspar Brekenfeld, Jens Fiehler, Christian Gerloff, Götz Thomalla, the GSR-ET-Investigators, C. Gerloff, J. Fiehler, G. Thomalla, A. Alegiani, Boeckh-Behrens, Silke Wunderlich, Ulrike Ernemann, Sven Poli, Eberhard Siebert, Christian H. Nolte, Sarah Zweynert, Georg Bohner, Alexander Ludolph, Karl-Heinz Henn, Jan Hendrik Schäfer, Fee Keil, Joachim Röther, Bernd Eckert, Jörg Berrouschot, Albrecht Bormann, Franziska Dorn, Gabor Petzold, Christoffer Kraemer, Hannes Leischner, Christoph Trumm, Steffen Tiedt, Lars Kellert, Martina Petersen, Florian Stögbauer, Michael Braun, Gerhard F. Hamann, Klaus Gröschel, Timo Uphaus, Arno Reich, Omid Nikoubashman, Peter Schellinger, Jan Borggrefe, Jörg Hattingen, Jan Liman, Marielle Ernst

**Affiliations:** 1 Department of Neurology, University Medical Center Hamburg-Eppendorf, Martinistrasse 52, 20246 Hamburg, Germany; 2 Department of Neuroradiological Diagnostics and Intervention, University Medical Center Hamburg Eppendorf, Hamburg, Germany

**Keywords:** Stroke, Thrombectomy, Real world, Health-related quality of life

## Abstract

**Background:**

Patients with a left (LHS) or right hemispheric stroke (RHS) differ in terms of clinical symptoms due to lateralization of specific cortical functions. Studies on functional outcome after stroke and endovascular thrombectomy (EVT) comparing both hemispheres showed conflicting results so far. The impact of stroke laterality on patient-reported health-related quality of life (HRQoL) after EVT has not yet been adequately addressed and still remains unclear.

**Methods:**

Consecutive stroke thrombectomy patients, derived from a multi-center, prospective registry (German Stroke Registry) between June 2015 and December 2019, were included in this study. At 90 days, outcome after EVT was assessed by the modified Rankin scale (mRS) and HRQoL using the European QoL-five dimensions questionnaire utility-index (EQ-5D-I; higher values indicate better HRQoL) in patients with LHS and RHS. Adjusted regression analysis was applied to evaluate the influence of stroke laterality on outcome after EVT.

**Results:**

In total, 5683 patients were analyzed. Of these, 2953 patients (52.8%) had LHS and 2637 (47.2%) RHS. LHS patients had a higher baseline NIHSS (16 vs. 13, p < 0.001) and a higher ASPECTS (9 vs. 8, p < 0.001) compared to RHS patients. Among survivors, patients with LHS less frequently had a self-reported affected mobility (p = 0.037), suffered less often from pain (p = 0.04) and anxiety/depression (p = 0.032) three months after EVT. After adjusting for confounders (age, sex, baseline NIHSS), LHS was associated with a better HRQoL (ß coefficient 0.04, CI 95% 0.017–0.063; p = 0.001), and better functional outcome assessed by lower values on the mRS (ß coefficient − 0.109, CI 95% − 0.217–0.000; p = 0.049).

**Conclusions:**

Ninety days after EVT, LHS patients have a better functional outcome and HRQoL. Patients with RHS should be actively assessed and treated for pain, anxiety and depression to improve their HRQoL after EVT.

## Introduction

Endovascular thrombectomy (EVT) has become standard of care for anterior circulation stroke in patients with large vessel occlusion irrespective of stroke lateralization. Strokes affecting the left and right hemisphere, however, are different with regards to acute clinical symptoms and outcome. Lateralization of specific cortical functions, e.g., language or spatial perception, results in important clinical differences between the hemispheres. In line with this, previous studies have demonstrated that patients with right hemispheric stroke (RHS) are less frequently recognized as having a stroke, present later to hospital, are less likely to receive intravenous thrombolysis therapy (IVT) and show a worse functional recovery compared to patients suffering from left hemispheric stroke (LHS) [1–3].

Moreover, imaging studies revealed that RHS may comprise a substantial and similar infarct size compared to LHS, while having a lower National Institutes of health Stroke Scale Score (NIHSS), thus, indicating that the NIHSS is biased towards LHS and presumably underpowered for assessing the clinical severity of RHS adequately [4–6].

However, studies on functional outcome, commonly assessed by the modified Rankin Scale (mRS), comparing RHS and LHS have provided conflicting evidence so far [7–9]. To our knowledge, the impact of hemispheric lateralization on quality of life in stroke patients after EVT has not yet been adequately addressed.

Thus, the objective of our study was to determine whether (a) outcome of stroke patients after EVT, assessed by the modified Rankin Scale (mRS) and self-reported HRQoL, differ between LHS and RHS patients and (b) stroke laterality has an influence on these outcome parameters after EVT in a large, representative, multi-center patient cohort of clinical practice.

## Methods

### Patients and data collection

In this study, patients from the German Stroke Registry-Endovascular Treatment (GSR-ET; ClinicalTrials.gov, Identifier: NCT03356392), enrolled between June 2015 and December 2019, were analyzed. The GSR-ET is an ongoing prospective, multicenter registry which comprises both university and community hospitals in Germany. This registry includes data from consecutive acute ischemic stroke patients with proximal large vessel occlusion of the anterior and posterior circulation treated with EVT. The study design and major findings have already been reported elsewhere [10, 11].

The decision for EVT was made at the physicians ‘decretion, interdisciplinary between a vascular neurologist and an interventional neuroradiologist. The neuroradiologists performed the interventions in accordance with the institutional guidelines. Ninety days after stroke thrombectomy, functional outcome and HRQoL were assessed by a standardized telephone interview or face-to-face visit by a well-trained investigator, who was blinded to patients’ variables.

The study was approved by all responsible ethics committees of the participating sites. The patients themselves or their proxies gave written informed consent. Consent was waived if patients died before consent could be obtained or lacked the capacity to give consent and no proxy was available.

### Health-related quality of life assessment

The three level European QoL-five dimensions (EQ-5D-3L) questionnaire, as an established instrument to assess quality of life, was applied to evaluate the self-reported HRQoL in this patient cohort. A detailed description is provided elsewhere [12]. Briefly, the EQ-5D-3L comprises the five health domains mobility, selfcare, usual activities, pain/discomfort and anxiety/depression. Patients may choose one of the three different response options for each of these five dimensions: no complaints, some complaints or extreme complaints. In total, 243 (3^5^) health states, and thus, 243 different indices can be calculated to evaluate the HRQoL [13]. Previously, this EQ-5D utility index (EQ-5D-I) was established by the time trade off method and is based on the country-specific value set of the UK population for this patient cohort, since a German-specific value set for the EQ-5D questionnaire is still unavailable and both countries are commonly assumed to be comparable [13]. As per instrument validation, a zero score is assigned to patients who died. Due to the time trade off method negative index values may also be achieved and are interpreted as situations in which patients might evaluate their health state worse than death. In our patient population, the minimum negative value was -0.594. Higher index values reflect better HRQoL, with the value “1” as the best health status. The patients themselves, their proxies or health care providers, if patients lacked capacity to respond, provided the EQ-5D information.

### Statistical analysis

Continuous variables are reported as median and interquartile range (IQR) or mean ± standard deviation (SD). Categorical variables are provided as proportions. Between group comparisons (LHS vs. RHS) for continuous variables were performed by Mann–Whitney-U-tests, for catagorical variables by chi-square or Fisher’s Exact tests. Of note, one part of the present study population has already been analyzed in terms of HRQoL after stroke thrombectomy, and the results were published recently [12].

Multivariate linear and binary logistic regression analyses assessed the influence of stroke laterality on outcome with the mRS and the EQ-5D-I, and with mortality (dichotomized to mRS 6 vs. 1–5) at 90 days as dependent variables. The analyses were adjusted by important clinical baseline variables: age, sex and NIHSS on admission. The resulting ß coefficients and odds ratios (OR) with 95% confidence intervals (CI) and p values are reported. P values < 0.05 were considered statistically significant. The statistical analysis was performed using SPSS (Version 25.0; IBM, Armonk, New York).

## Results

### Patient cohort

**Fig. 1:**
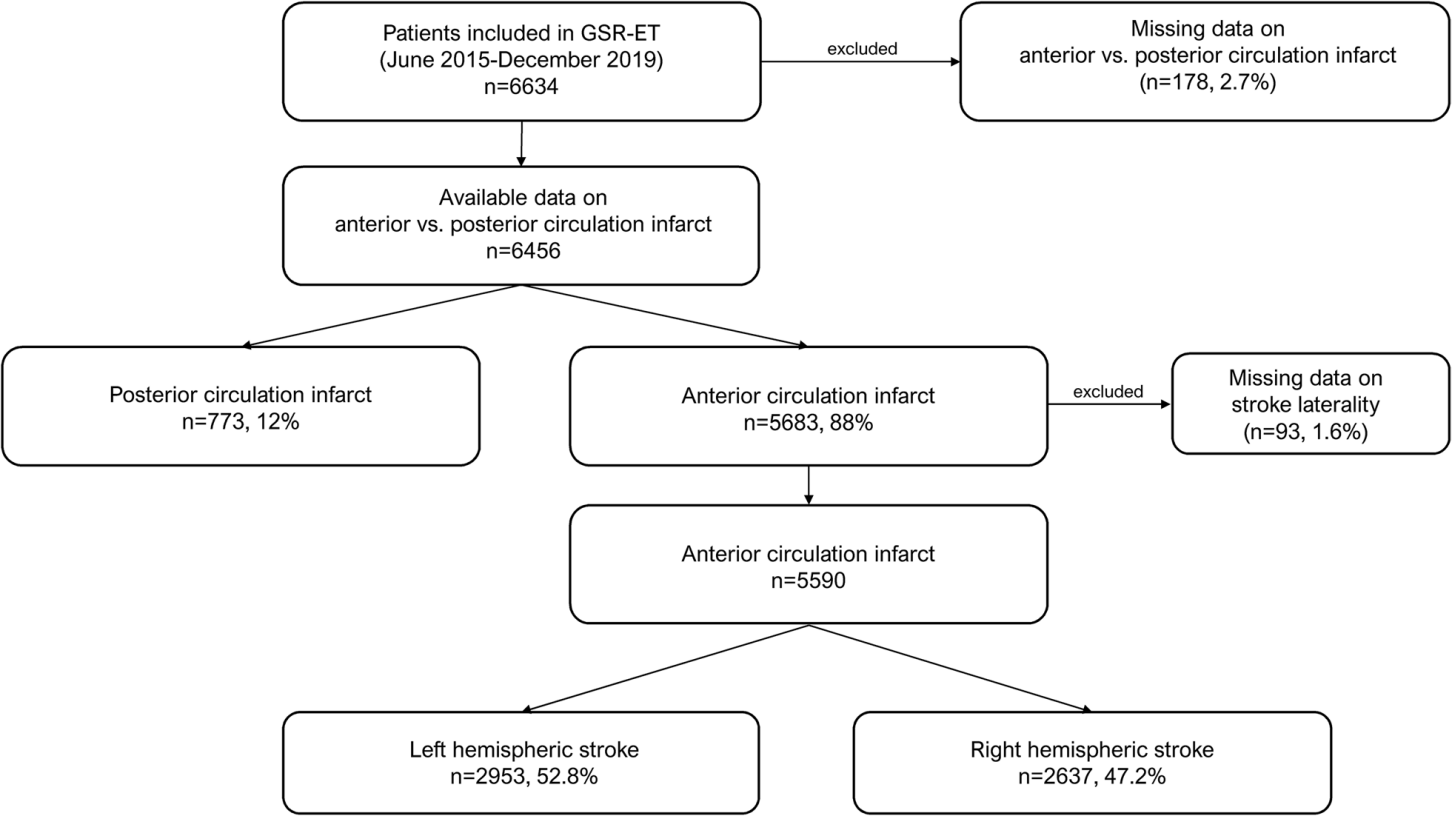
Flowchart of patients included in this subanalysis of the GSR-ET. In total, n = 6634 patients were enrolled in the GSR-ET between June 2015 and December 2019. After excluding subjects with missing data n = 5590 patients were included in the present study, whereas 53% of these had a LHS and 47% a RHS

In total, n = 6634 patients were enrolled in the registry during the study period (Fig. 1). Data on infarct location were available in n = 6456 patients, with n = 5683 (88%) subjects suffering from anterior circulation infarcts. In n = 93 (1.6%) data on stroke laterality were missing, leaving n = 5590 patients for this analysis. Of these, n = 2953 patients (52.8%) had LHS, and n = 2637 (47.2%) patients had RHS (Table 1). Patients with LHS had a higher stroke severity on admission assessed by the NIHSS (16 vs. 13, p < 0.001) and a higher Alberta Stroke Program Early CT Score (ASPECTS) (9 vs. 8, p < 0.001) compared to patients with RHS. The time elapsed from symptom onset to groin puncture and recanalization were shorter in LHS patients (189.5 vs. 200 min, p = 0.004 and 236 vs. 248 min, p = 0.012, respectively) than in RHS subjects. LHS patients less frequently had any intracranial hemorrhage (ICH) 24 h after intervention (11.1 vs. 13.2%, p = 0.008).

**Table 1:** Descriptive statistics of LHS (n = 2953) compared to RHS (n = 2637) patients

	**LHS** **N = 2953**	**RHS** **n = 2637**	**P value**
Age—median (IQR)	76 (65–82)	76 (66–83)	0.911
Sex (female)—n (%)	1528/2950 (51.8)	1376/2637 (52.2)	0.397
Living status before admission n = 5232			0.689
Home	2435/2766 (88.0)	2169/2466 (88.0)	
Nursing at home	128/2766 (4.6)	105/2466 (4.3)	
Nursing home	203/2766 (7.3)	192/2466 (7.8)	
Pre-existing comorbidities			
Atrial fibrillation n = 5280	1213/2789 (43.5)	1064/2491 (42.7)	0.294
Arterial hypertension n = 5284	2136/2788 (76.6)	1961/2496 (78.6)	0.048
Dyslipidemia n = 5273	1104/2784 (39.7)	982/2489 (39.5)	0.452
Diabetes Mellitus n = 5283	603/2788 (21.6)	561/2495 (22.5)	0.237
Pre-stroke mRS > 1—n (%)	560/2739(20.4)	525/2436 (21.6)	0.173
NIHSS on admission—median (IQR)	16 (10–20) n = 2792	13 (9–17) n = 2481	< *0.001*
Mothership—n (%)	1685/2953 (57.1)	1473/2637 (55.9)	0.190
ASPECTS—median (IQR)	9 (7–10) n = 2626	8 (7–10) n = 2354	< *0.001*
IVT—n (%)	1480/2938 (50.4)	1373/2618 (52.4)	0.065
Anesthesia			*0.002*
beginning with local anesthesia change to general anesthesia	106/2847 (3.7)	84/2528 (3.3)	
conscious sedation	746/2847 (26.2)	774/2528 (30.6)	
general anesthesia	1995/2847 (70.1)	1670/2528 (66.1)	
mRS 24 h—median (IQR)	5 (3–5) n = 2553	4 (3–5) n = 2276	*0.006*
NIHSS 24 h—median (IQR)	12 (5–20) n = 2530	10 (4–17) n = 2219	< *0.001*
mRS discharge median (IQR)	4 (2–5) n = 2763	4 (2–5) n = 2445	*0.018*
NIHSS discharge—median (IQR)	6 (2–15) n = 2389	5 (2–12) n = 2136	*0.002*
Stroke etiology—n (%)			0.156
Artherosclerosis	626/2953 (21.2)	611/2637 (23.2)	
Cardioembolic stroke	1523/2953 (51.6)	1284/2637 (48.7)	
Stroke of undetermined etiology	461/2953 (15.6)	435/2637 (16.5)	
Stroke of other determined etiology	120/2953 (4.1)	95/2637 (3.6)	
Dissection	44/2953 (1.5)	46/2637 (1.7)	
Length of stay days—median (IQR)	9 (5–14) n = 2798	9 (5–13) n = 2485	0.281
*Workflow times*			
Symptom onset to groin puncture (min)—median (IQR)	189.5 (135–265) n = 1634	200 (145–275) n = 1512	*0.004*
Symptom onset to recanalization (min)——median (IQR)	236 (179–318) n = 1455	248 (190–328.25) n = 1354	*0.012*
*Outcome parameters*			
mTICI 2b/3—n (%)	2464/2921 (84.4)	2221/2610 (85.1)	0.234
Any ICH 24 h– n (%)	327/2953 (11.1)	348/2637 (13.2)	*0.008*
mRS 0–2 at 90 days—n (%)	885/2406 (36.8)	828/2191 (37.8)	0.250
mRS 5–6 at 90 days—n (%)	916/2406 (38.1)	801/2191 (36.6)	0.152
mRS 6 at 90 days—n (%)	726/2406 (30.2)	590/2191 (26.9)	*0.008*
mRS at 90 days—median (IQR)	4 (1–6) n = 2406	4 (1–6)	0.139
EQ-5D-I—mean (± SD)	0.566 (± 0.467)	0.556 (± 0.462)	0.308

LHS, Left hemispheric stroke; RHS, Right hemispheric stroke; IQR, Interquartile range; mRS, modified Rankin Scale; NIHSS, National Institutes of Health Stroke Scale; ASPECTS, Alberta Stroke Program Early CT Score; IVT, intravenous thrombolysis; mTICI, modified Thrombolysis in Cerebral Infarction Score; ICH, Intracerebral hemorrhage; EQ-5D-I, European Quality of Life-five dimensions questionnaire utility-index; SD, standard deviation

### Functional outcome assessed by the mRS

**Fig. 2:**
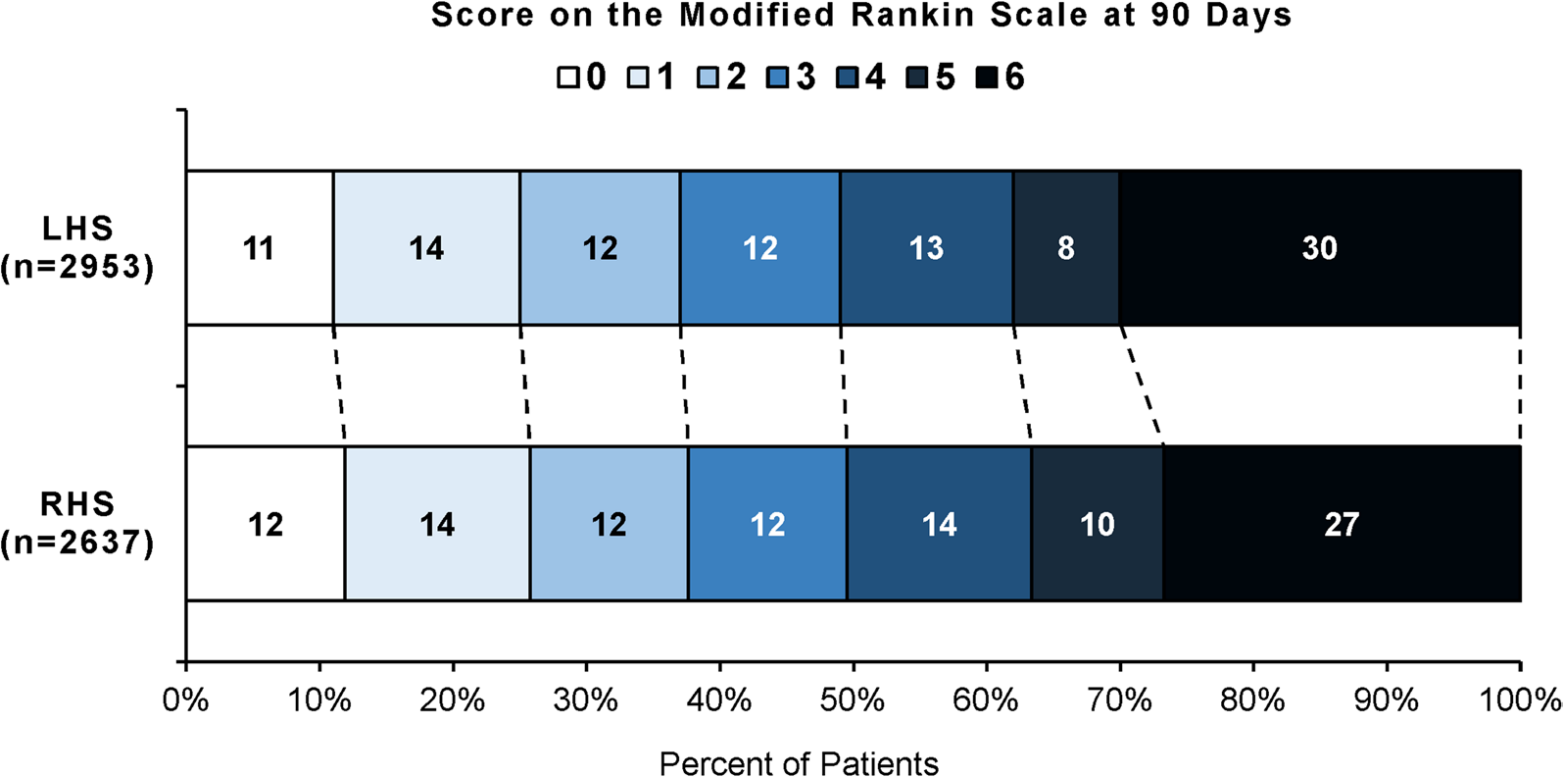
The distribution of the mRS scores at 90 days according to the affected hemisphere. The rate of functional independence (mRS 0–2) and death or dependency (mRS 5–6) showed no difference between LHS and RHS patients. In unadjusted analysis, a 3.3% higher mortality rate was found in LHS compared to RHS patients 90 days after stroke thrombectomy (30.2 vs. 26.9%, p = 0.008)

The distribution of the mRS scores at 90 days of both LHS and RHS patients is displayed on Fig. 2. The rate of functional independence (mRS 0–2) and death or dependency (mRS 5–6) did not differ between both patient subgroups. In unadjusted analysis, LHS patients had 3.3% higher mortality rate 90 days after stroke thrombectomy than RHS patients (30.2 vs. 26.9%, p = 0.008). After adjusting for confounding variables, hemispheric lateralization showed no association with mortality at 90 days anymore (OR 1.011, CI 95% 0.876–1.167; p = 0.879).

**Fig. 3:**
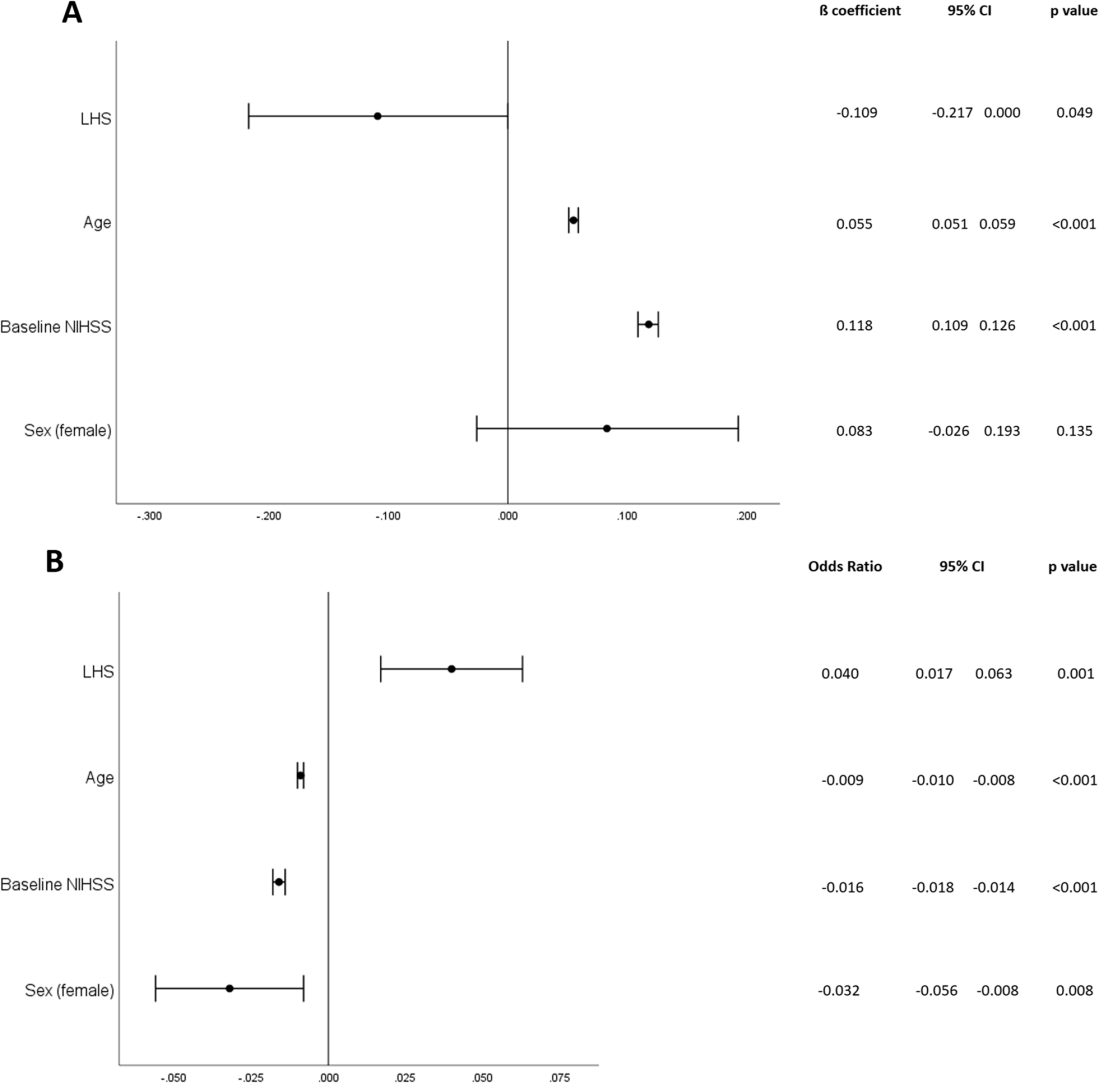
Forrest plot showing the impact of stroke laterality on outcome assessed by the mRS (**A**) and by the EQ-5D-I **B** 90 days after EVT. After adjusting for the confounding variables (age, sex and baseline NIHSS), LHS was associated with a better functional outcome (ß coefficient − 0.109, CI 95% − 0.217–0.000; p = 0.049) (Fig. 3A) and strongly associated with a better HRQoL (ß coefficient 0.04, CI 95% 0.017–0.063; p = 0.001) (Fig. 3B)

In adjusted analyses, LHS was associated with a better functional outcome (ß coefficient -0.109, CI 95% − 0.217–0.000; p = 0.049) (Fig. 3A).

### Health-related quality of life assessed by EQ-5D-I

In adjusted analyses, LHS was associated with a better HRQoL (ß coefficient 0.04, CI 95% 0.017–0.063; p = 0.001) (Fig. 3B).

**Fig. 4:**
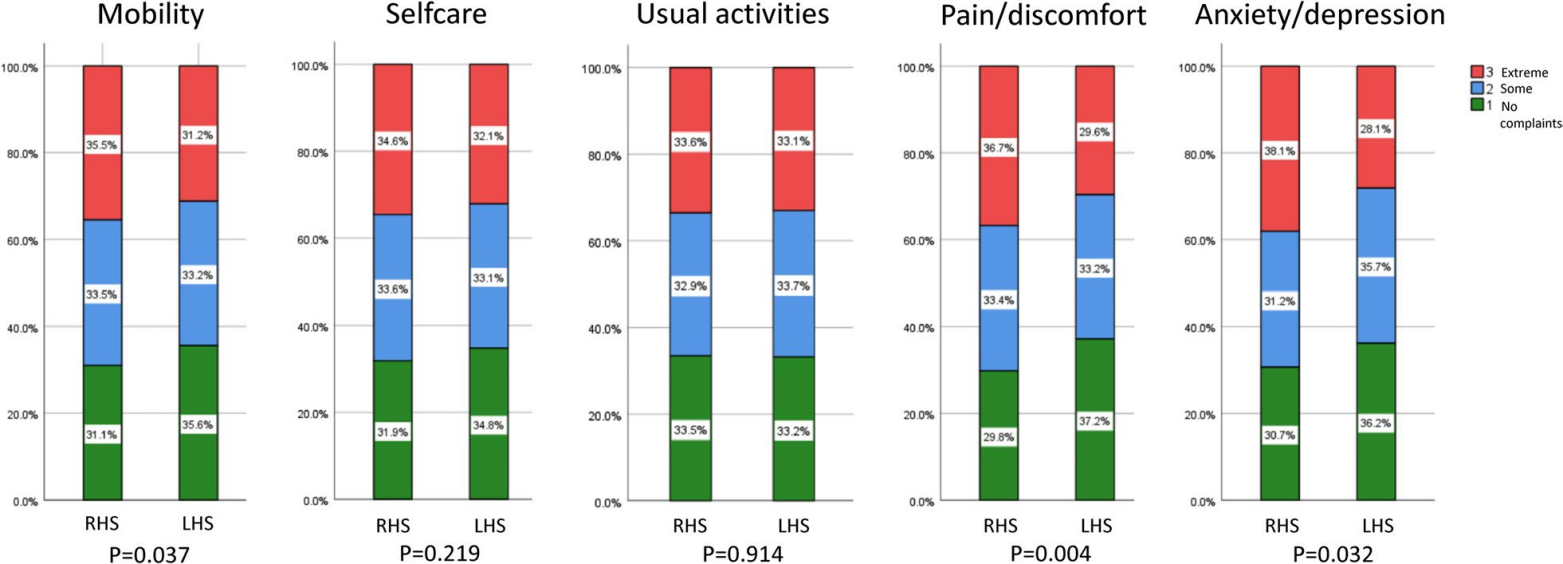
Health-related quality of life 90 days after EVT between patients with LHS and RHS. The distribution of the EQ-5D 3L results in LHS compared to RHS patients is displayed. Patients with LHS had less often mobility constraints (p = 0.037), suffered less frequently from pain (p = 0.04) and anxiety/depression (p = 0.032) 90 days after EVT compared to RHS subjects. The patient responses in the dimensions selfcare (p = 0.219) and usual activities (p = 0.914) showed no differences between both hemispheres. GSR-ET, German Stroke Registry-Endovascular Treatment; LHS, Left hemispheric stroke; RHS, Right hemispheric stroke; mRS, modified Rankin Scale; EQ-5D-I, European Quality of Life-five dimensions questionnaire utility-index; EVT, endovascular treatment; NIHSS, National Institutes of Health Stroke Scale

Patients with LHS less frequently had a self-reported affected mobility (p = 0.037), suffered less often from pain (p = 0.04) and anxiety/depression (p = 0.032) three months after stroke thrombectomy compared to RHS subjects. The patient responses in terms of the dimensions selfcare (p = 0.219) and usual activities (p = 0.914) did not differ between the hemispheres (Fig. 4).

## Discussion

In the present study, which included stroke thrombectomy patients from a large, prospective, multi-center and industry-independent registry, we provide *real world* data on the impact of stroke laterality on functional outcome and HRQoL, and detailed information about different health dimensions after EVT in patients with LHS compared to RHS.

In this study cohort of clinical practice, we found an effect of hemispheric lateralization on functional outcome assessed by mRS. In addition, we observed a highly significant effect of stroke laterality on HRQoL.

Most of the trials on stroke and thrombectomy assessed functional outcome by the mRS which mainly reflects the physical disability of a patient. Previous studies already demonstrated that the impact of stroke laterality on functional outcome after stroke and thrombectomy is still inconclusive [7–9]. In line with this, the association of hemispheric lateralization only showed a ß coefficient of − 0.109 (CI 95% − 0.217–0.000; p = 0.049) in our patient cohort.

However, previous studies on stroke and thrombectomy have suggested to assess outcome and effectiveness of medical treatment by additional scales beside the mRS, such as patient self-reported outcomes. In particular, HRQoL is a valuable complementary outcome measure to evaluate outcome after stroke and EVT, as already applied and stated by several studies [12, 14, 15]. The European Stroke Organization has also prioritized the use of patient-reported outcomes in research studies to develop specific targets in stroke rehabilitation and facilitate the recovery process [16].

One possible explanation for the better HRQoL in LHS patients is that recovery might be protracted in RHS patients due to non-dominant hemisphere symptoms such as depression, apathy and amotivation [3, 7]. Furthermore, LHS patients tend to remain longer in rehabilitation [17] which may also account for the better HRQoL in this subgroup of patients. A lower rehabilitation potential of RHS patients due to neglect has also been discussed [18, 19].

In fact, in our study population RHS patients more frequently suffered from pain/discomfort and anxiety/depression, and perceived higher mobility constraints than LHS patients at 90 days. Thus, RHS patients might be scored well on available functional assessment scores while still being extremely disabled by these factors. Thus, our findings provide valuable additional data for targeted rehabilitation therapies after EVT, in particular for patients with RHS. In consequence, our results suggest that patients with RHS should be actively assessed and treated for pain, anxiety and depression beside physical disability after EVT to improve their quality of life.

In line with our results, a previous study found a significant association of depression with stroke lesions of the right hemisphere [3]. Furthermore, since in LHS patients speech disabilities are in the foreground, they might perceive mobility impairments less than RHS subjects. Moreover, patients with RHS suffer more frequently from the pusher-syndrome (lateropulsion) after stroke [20] which might also account for a worse perception of mobility. Interestingly, imaging studies revealed an increased activation of the right-sided insula in the presence of neuropathic pain [21, 22], indicating a strong association of right hemispheric lesions with the perception of pain, consistent with our findings.

In between-group comparisons, we discovered a higher baseline NIHSS in LHS patients compared to RHS patients. Thus, we confirmed previous findings, as several studies found similar results after stroke and thrombectomy [7–9, 23]. The difference in the NIHSS between the hemispheres might be explained by a structural inherent bias of the NIHSS itself. In particular, the NIHSS is biased towards LHS, as the NIHSS gives more weight to language, attributable to left hemispheric lesions, than to hemispatial neglect, a hallmark of RHS [6].

Notably, patients with RHS were shown to have a comparable infarct volume as LHS [4] despite scoring less on the NIHSS. This indicates that the NIHSS systematically underestimates stroke severity in RHS patients [4]. The different weighting in scoring the NIHSS for both hemispheres may impact on treatment decision that rely on NIHSS tresholds. In fact, RHS patients were shown to receive less frequently IVT than LHS subjects [7]. It was suggested that a combination of late diagnosis, delayed admission to hospital and the use of stroke severity scales biased towards the left hemisphere may have induced this imbalance of treatment [18, 19]. However, we did not find different IVT rates between the hemispheres in our patient cohort of clinical practice.

The differences in clinical symptoms depending on the side of lesion may, indeed, affect awareness and recognition of stroke. Since sudden speech disturbances are more apparent than perceptual deficits, RHS patients are more likely to be later diagnosed as a stroke, and, in consequence, present later to hospital [1]. This is in line with our findings showing longer workflow times in RHS patients of our study cohort. Interestingly, the ASPECT score was found to be lower in RHS patients, potentially explained by the deferred admission [1]. Correspondingly, the higher rate of any ICH 24 h after intervention in RHS patients might be attributed to the higher extent of early ischemic changes on brain imaging in RHS patients on admission.

There are some factors that may limit our findings. Our study provides a short-term follow up period of three months after stroke thrombectomy for HRQoL measurements. Long-term assessments may provide additional clues about HRQoL. Furthermore, in cases who lacked capability the questionnaire was completed by proxies. This might reduce the validity of the responses, since over- or underestimation of QoL might occur. However, we believe that this limitation did not significantly confound our findings, since agreement between caregivers and patient self-reports have been demonstrated to be reasonable [24].

## Conclusions

This study represents real world experience and provides data from a full spectrum of patients with acute ischemic stroke treated with EVT over a broad range of stroke severity. Stroke laterality had an influence on functional outcome assessed by the mRS, and showed a clear impact on HRQoL 90 days after EVT. Our findings reinforce that assessment of HRQoL as a complimentary outcome measure is of paramount importance, since it enables an identification of non-motor determinants affecting HRQoL, such as pain, anxiety and depression. In particular, in patients with RHS rehabilitation needs for these factors should not be underestimated, but rather actively assessed and specifically targeted in rehabilitation therapies to improve their quality of life.

## Data Availability

The datasets used and analysed during the current study are available from the corresponding author on reasonable request.

## Abbreviations

LHS: Left hemispheric stroke
RHS: Right hemispheric stroke
HRQoL: Health-related quality of life
GSR-ET: German Stroke Registry-Endovascular Treatment
EQ-5D-I: European quality of life-five dimension questionnaire utility index
EQ-5D-3L: Three level European quality of life-five dimensions questionnaire
